# Virtual clinic for young people with type 1 diabetes: a randomised wait-list controlled study

**DOI:** 10.1186/s12902-023-01516-x

**Published:** 2023-11-22

**Authors:** Janeth Leksell, Eva Toft, Jessica Rosman, Jan W. Eriksson, Johan Fischier, Anna Lindholm-Olinder, Andreas Rosenblad, Elisabet Nerpin

**Affiliations:** 1 Department of Medical Sciences, Clinical Diabetology and Metabolism, Uppsala University, Akademiska sjukhuset, Uppsala, SE-75185 Sweden; 2 School of Education, Health and Social Studies, Dalarna University, Falun, Sweden; 3 Department of Medicine, Karolinska Institutet, Huddinge, Stockholm, Sweden; 4 Diabetes Unit, Ersta Hospital, Stockholm, Sweden; 5 Diabetes Unit, Karolinska University hospital, Stockholm, Sweden; 6 Department of Clinical Science and Education, Karolinska Institutet, Södersjukhuset, Stockholm, Sweden; 7 Department of Neurobiology, Care Sciences and Society, Division of Family Medicine and Primary Care, Karolinska Institutet, Stockholm, Sweden; 8 Department of Statistics, Uppsala University, Uppsala, Sweden; 9 Department of Medical Sciences, Respiratory Medicine, Allergy and Sleep, Uppsala University, Uppsala, Sweden; 10 Department of Medical Sciences: Clinical Physiology, Uppsala University, Uppsala, Sweden

**Keywords:** Type 1 Diabetes, Virtual Diabetes clinic, Young adults, Glycaemic control, Treatment satisfaction, Quality of life

## Abstract

**Background:**

The transition from paediatric to adult care for young adults with type 1 diabetes poses unique challenges. Virtual diabetes clinics using smartphone applications offer a promising approach to support self-management and enhance communication with healthcare providers. The primary objective of this study was to evaluate the effects of a virtual diabetes clinic on glycaemic control, treatment satisfaction, and quality of life among young adults diagnosed with type 1.

**Methods:**

79 participants with type 1 diabetes aged 18–25 years were included in a prospective, single-centre, randomised, wait-list controlled trial. Participants were randomly assigned to either the intervention group or the wait-list control group. The intervention group received instant access to a virtual care platform called Vista Dialog, which facilitated real-time communication between patients and healthcare providers. Glycosylated haemoglobin (HbA1c) levels, time in range (TIR), time below range (TBR), diabetes treatment satisfaction, and quality of life were assessed at baseline and after 6 months.

**Results:**

Baseline characteristics were similar between the intervention and control groups, except for education level, where there was a skewed distribution between the groups (the intervention group had a lower education level). At the 6-month follow-up, there were no significant differences in HbA1c levels, TIR, TBR, or diabetes treatment satisfaction between the two groups. However, the intervention group demonstrated a significant decrease in the burden on physical health compared with the control group, indicating an improved quality of life.

**Conclusions:**

The implementation of a virtual diabetes clinic using the Vista Dialog platform did not result in significant improvements in glycaemic control or treatment satisfaction compared with usual care. However, it did show potential benefits in terms of reducing the burden on physical health and improving quality of life in young adults with type 1 diabetes. Further research is needed to explore the long-term effects and optimal use of virtual clinics in diabetes management.

**Trial registration:**

ISRCTN number: 73,435,627 (registration date: 23/10/2019): 10.1186/ISRCTN73435627. The performance and results of this trial adhere to the guidelines outlined in the CONSORT 2010 (Consolidated Standards of Reporting Trials) recommendations.

## Background

Diabetes is a chronic disease that poses a significant health risk, with potentially severe complications, including premature death [1]. Managing diabetes is a constant struggle with balancing blood sugar levels, diet, exercise, and other demands [2]. Patients with diabetes often experience feelings of fear, shame and isolation, but also report feeling courage and support from family and friends with diabetes [3].

Sweden has one of the highest percentages of children with type 1 diabetes in the world [4], and it is considered one of the most common chronic diseases in this group. In Sweden, the transition of adolescents with type 1 diabetes from pediatric care to adult care occurs at the age of 18. The transition from youth to adulthood can be a critical period in life. If the young adult must also deal with a chronic illness such as type 1 diabetes, this creates additional demands [5]. The transition from paediatric to adult care is often associated with higher blood sugar levels, intertwined with physiological and psychological changes, which may have an impact on diabetes self-management and diabetes-related outcomes such as glycosylated haemoglobin (HbA1c) levels [5–7].

Advanced treatment technologies, such as pumps and sensors, have created additional demands regarding care, education, and support for patients with type 1 diabetes and their families [8]. In Sweden, the proportion of children under the age of 18 who use continuous glucose monitoring (CGM) is 97%. For adults, the figure stands at 93%. The roles and responsibilities of healthcare professionals (HCPs) have also evolved, with person-centred care replacing the conventional hierarchical approach [9–11]. As a result, the question arises whether the diabetes care currently offered to patients is optimal. Telemedicine, such as smartphone applications (‘apps’), could be a low-cost intervention to support diabetes self-management [12]. Patients can communicate with healthcare providers from the comfort of their own home, with frequent contact if needed [13, 14].

Younger individuals are accustomed to using advanced technology such as insulin pumps and continuous or rapid glucose monitoring systems, which provide detailed information that can be used for online consultation with healthcare providers. This technology enables distance diabetes care with detailed analyses and recommendations. Virtual diabetes clinics via smartphone apps can be a way to support self-management of diabetes. Such clinics enable patients to communicate with healthcare providers, diabetes nurses or doctors, which can create security for the patients.

Past studies have suggested that it is crucial to evaluate additional health parameters alongside HbA1c and to strengthen the methodological rigor of future investigations [15]. Additionally, it has been noted that individuals with type 1 diabetes frequently face psychosocial challenges [5–7]. Meta-analyses and systemic reviews of randomised controlled trials (RCTs) have shown that telemedicine and telemonitoring interventions can be effective alternative methods for delivering healthcare and improve communication with persons with type 1 and type 2 diabetes [15–17]. The results of the meta-analyses suggest that interventions using virtual elements to promote glycaemic control have a significant impact on HbA1c levels in individuals with type 2 diabetes, but do not appear to have a similar effect in people with type 1 diabetes [15, 18, 19].

Furthermore, young adults who have type 1 diabetes make up a population that is hard to reach and often have deteriorated HbA1c levels. Curiously, there is currently a lack of virtual RCTs evaluating young adults with type 1 diabetes, despite the potential benefits and increasing use of virtual trials in other areas of research.

### Study aim

This study aims to assess the effect of a virtual diabetes clinic on glycaemic control, treatment satisfaction, and quality of life in a specific population of young adults between the ages of 18 and 25 diagnosed with type 1 diabetes.

## Method

### Study design

**Fig. 1:**
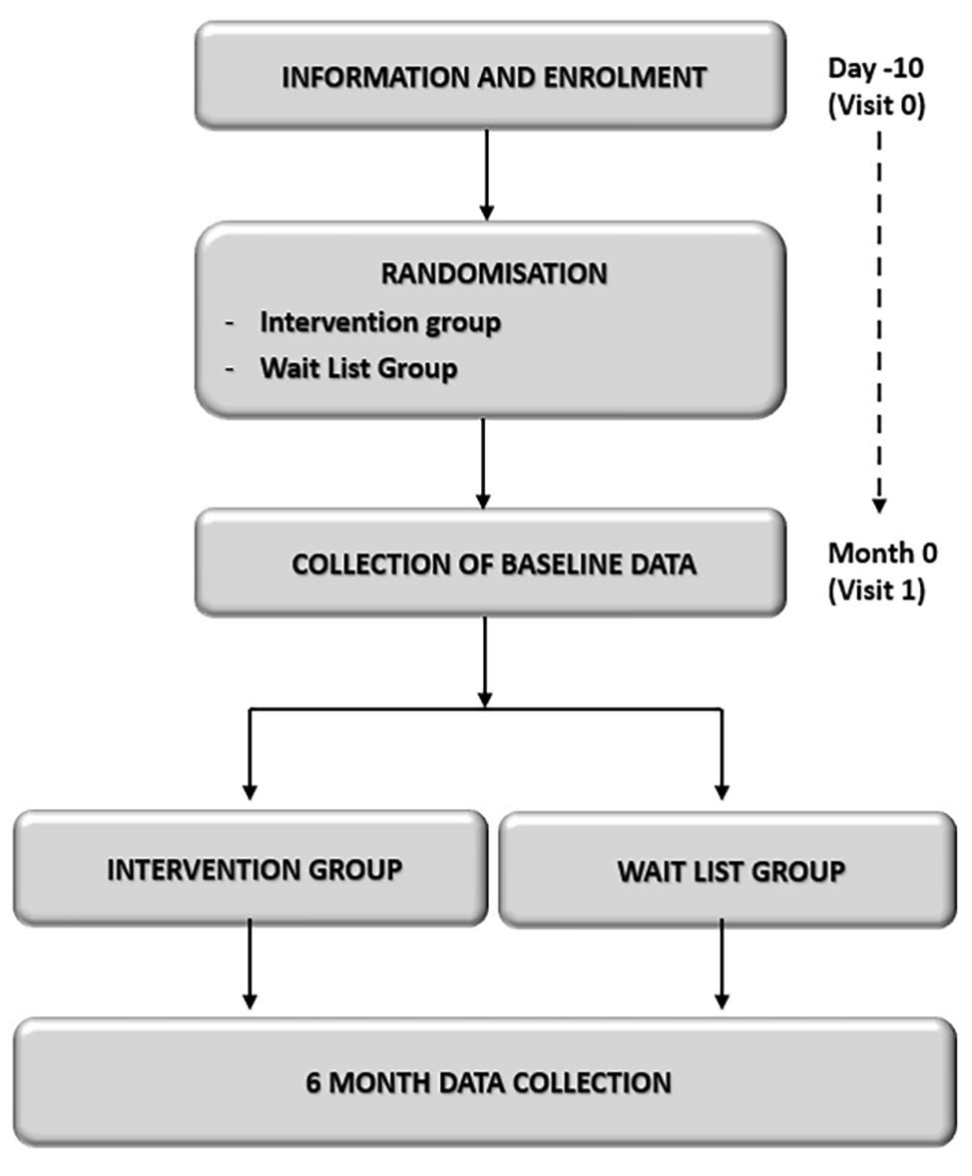
Flowchart of the trial

The study design and methods of the present study have been described in detail in a previously published study protocol [20]. Briefly, the study was conducted from 1th January 2019 to the end of May 2022, a total of 79 participants with type 1 diabetes were randomly assigned to a prospective, single-centre, randomised, wait-list controlled trial with a duration of 6 months (trial registration ISRCTN number: 73,435,627 (registration date: 23/10/2019); 10.1186/ISRCTN73435627). with balanced randomisation (1:1). The study implemented the CONSORT (Consolidated Standards of Reporting Trials) statement as a framework for reporting and conducting the research [21]. Figure 1 presents a comprehensive flowchart outlining the process of participant enrollment, intervention allocation, and follow-up in a visual format.

### Participants and recruitment

All participants had type 1 diabetes, were registered at a single hospital in Stockholm, Sweden, and were identified in the diabetes clinic’s patient register by hospital HCPs. The inclusion criteria of the study were access to a smartphone or computer, duration of diabetes for more than 1 year and an age of 18–25 years. Participants who met any of the following criteria were excluded from the study: diagnosis of severe depression, eating disorder, or other significant mental illness, history of alcohol or drug abuse, or presence of severe complications related to diabetes. The diabetes nurse and/or physician made the decision on whether each diabetes patient had the compliance required to participate in the study. All individuals received verbal and written information about the study before inclusion. Originally, our intention was to include 100 participants in the study. However, the widespread development of Covid-19 within society presented obstacles that hindered the recruitment of study subjects.

### Randomisation and intervention

The nurses at the clinic were responsible for including individuals in the study. Once informed consent was obtained from the participants, they were randomly assigned to either the intervention group or the wait-list control group. The randomization process involved the use of sealed envelopes containing randomized cards. The sealed envelopes were assembled by an impartial individual with no involvement in either the process of patient inclusion or their subsequent care.

All materials were coded with consecutive numbers and the code list was kept in a locked fireproof cabinet at the hospital. After inclusion, a first data collection was performed in both groups. The intervention group got immediate access to the virtual clinic (Fig. 1).

### Vista dialog

Vista Dialog is a virtual care platform, managed via secure login, delivered via a smartphone app for patients and a web interface/portal for HCPs. The platform facilitates seamless real-time communication for participants, enabling them to engage in text message exchanges, schedule online appointments with diabetes specialist nurses, and initiate impromptu video meetings if the situation necessitates further discussion related to their ongoing text message conversations with the nurses. The patient can also upload data from their insulin pump and continuous glucose monitoring (CGM) system for review and discussion. This invites and enables patients to put forward their needs when they arise.

### Measures and data collection

Data collection for both clinical variables and psychometric measures was conducted exclusively at the diabetes clinic. Baseline assessments were carried out prior to the intervention, and outcome measurements were collected at both the baseline and 6-month follow-up time points. At baseline, comprehensive data encompassing sociodemographic information, including sex, age, living arrangements (whether living at home or in independent living), and education level, were collected to provide a descriptive profile of the participants. Furthermore, diabetes duration, age at onset of diabetes, and type of treatment such as multiple daily injections (MDI) or continuous subcutaneous insulin infusion (CSII). Moreover, height (m) and weight (kg) were measured by trained HCPs and used to calculate body mass index (BMI).

### Clinical variables

At the standard clinic appointments, the following data were collected:

- HbA1c (Afinion 2™ [Abbott, USA]) level was used to reflect the average plasma glucose level over the preceding 8–12 weeks.
- Time in range (TIR), the percentage of time that a person spends with their glucose levels in a targeted range (3.9–10 mmol/L), and time below range (TBR), the percentage of time that a person spends with their glucose levels at < 3.9 mmol/L. TIR and TBR were measured through real-time CGM (rtCGM)/intermittently scanned CGM (isCGM).
- The insulin dosage information was extracted and recorded using diabetes management software, such as Diasend®, which allows for data downloading and analysis.
- Daily insulin dose (collected where possible).

### Diabetes treatment satisfaction questionnaire

Diabetes Treatment Satisfaction Questionnaire, status version (DTSQs), was used to evaluate patients’ satisfaction with diabetes treatment interventions and developed and validated by Clare Bradley. This questionnaire is well-established for use in diabetes research [22].

The DTSQs consists of three areas with a total of eight questions. The first area includes six questions covering aspects of treatment satisfaction, such as ‘satisfaction with current treatment,‘ ‘flexibility,‘ ‘convenience,‘ ‘understanding of diabetes,‘ ‘recommend treatment to others,‘ and ‘willingness to continue.‘ These questions are rated on a 7-point scale from ‘very dissatisfied’ to ‘very satisfied,‘ with a maximum score of 36, indicating higher treatment satisfaction. The second and third areas consist of single questions each, related to experiences of hyperglycemia and hypoglycemia in the weeks leading up to the assessment. Each question is rated on a 7-point scale from 0 to 6 [22].

In summary, the DTSQs provides insights into treatment satisfaction, hyperglycemia and hypoglycemia experienced by patients in diabetes research studies.

### Check your health questionnaire

The validated questionnaire ‘Check your health’ consists of vertical scales (0–100 points) intended to screen for perceptions and experiences of physical and emotional health, social relationships and general quality of life [23]. The “Check your health” assessment also includes questions aimed at capturing the respondent’s perception of their physical and emotional health, social relationships, and overall quality of life in the hypothetical scenario of not having diabetes. Low scores reflect a diminished perception of health and quality of life, whereas high scores denote an elevated perception of health and quality of life. When there is a positive difference between two conditions (for example, reporting lower physical health without diabetes compared to with diabetes), the burden is considered as zero [23]. The difference between, for example, physical health with and without diabetes is defined as the physical burden of diabetes. The interpretation of the values is no burden (0), low burden (1-10), high burden (11–29) and very high burden (≥ 30). The Check your health questionnaire was used at baseline and after 6 months for both the intervention group and the control group.

### Statistical analyses

A power analysis was conducted to determine the sample size needed to detect a mean difference of 6 mmol/mol (standard deviation [SD] = 9) in HbA1c. To achieve a power of 80% with a significance level of 0,05 in a two-sided test. It determined that a minimum of 37 participants should be included in each group. Considering potential dropout rates, a total of 100 patients was planned to be enrolled in the study. Participants was randomly assigned to either experimental or control group with a 1:1 distribution as per a computer-generated randomisation.

Categorical data are described with frequencies and percentages, n (%), while ordinal, discrete, and continuous data are given as means with accompanying standard deviations (SDs). Tests of differences between baseline and 6-month follow-up within groups were performed using paired t-test for continuous data and the Wilcoxon signed-rank test with continuity correction for ordinal data, whereas tests of differences in changes from baseline to 6-month follow-up between intervention and control groups were performed using the Welch two-sample t-test for continuous data and the Wilcoxon rank-sum test with continuity correction for ordinal data. All statistical analyses were performed in R 4.1.2 (R Foundation for Statistical Computing, Vienna, Austria), with two-sided P-values < 0.05 considered statistically significant.

### Ethics

Prior to their inclusion in the study, informed consent was obtained from all participants. The study was conducted in accordance with the principles and all the subsequent amendments of the declaration of Helsinki. The Swedish Ethical Review Authority in Uppsala approved this study with diary number 2018–568 and diary number 2019–00133. Additionally, the clinical trial has been registered at ISRCTN under the number 73,435,627; 10.1186/ISRCTN73435627.

## Results

Baseline characteristics for the intervention and control groups are given in Table 1. The study included 79 participants, with 35 assigned to the intervention group and 44 to the control group. The average age (± SD) of the intervention group was 19.8 (± 1.8) years, while the control group had an average age of 20.7 (± 1.9) years. The duration of diabetes was similar in both groups, with an average of 10.7 (± 4.7) years for the intervention group and 10.5 (± 5.1) years for the control group. About half of the participants in each group were receiving pump treatment, while the other half received multiple doses. Of these participants, 36% had rtCGM, 61% had isCGM and 3% used capillary glucose measurements with a hand-held glucometer. There were no significant differences between the groups in terms of age, sex, diabetes duration, type of therapy (CSII/MDI) or HbA1c levels at baseline except for education level, where there was a skewed distribution between the groups (the intervention group had a lower education level) (Table 1).

**Table 1:** Baseline characteristics of the intervention (n = 35) and control (n = 44) groups

Variable	Intervention	Control
Female sex, n (%)	21 (60.0)	31 (70.5)
Age, mean (SD)	19.8 (1.8)	20.7 (1.9)
HbA1c, mean (SD)	62.8 (14.8)	58.8 (11.5)
Time in range, mean (SD), n = 76	45.9 (13.3)	49.7 (16.5)
Glucose monitoring n = 78		
− rtCGM n (%)	10 (29)	18 (41)
− isCGM n (%)	23 (68)	25 (57)
− hand-held glucometer n (%)	1 (3)	1 (2)
Years with diabetes, mean (SD)	10.7 (4.7)	10.5 (5.1)
Pump, n (%)	18 (51)	22 (50)
Multiple doses, n (%)	16 (49)	22 (50)
Education level, n (%)		
− Primary level	11 (31.4)	5 (11.4)
− Secondary level	22 (62.9)	35 (79.5)
− College / University	2 (5.7)	4 (9.1)

Note: NS, non-significant; SD, standard deviation. Missing value: Glucose monitoring intervention group n = 34, Time in range: intervention group n = 33 and control group n = 43

### Follow-up after 6 months

At the 6-months follow-up, 71 participants remained in the study, with 7 dropping out due to relocation for studies or work elsewhere and 1 for unknown reasons (2 in the intervention-, and 6 in the control group). Among the 33 individuals in the intervention group, 29 participants actively used one or more functions of the application, Table 2. Results for primary and secondary outcomes at baseline and 6-months follow-up for individuals with measurements at both time points are given in Table 3.

**Table 2:** Different usage of communication path and number of times

Different communication paths using the app n = 29	Number of users who have used respective function divided by the number of times.				
	0 times	1–3 times	4–6 times	7–9 times	≥ 10 times
Contact through chat n, (%)	5 (15)	16 (49)	10 (30)	0 (0)	2 (6)
Contact through video n, (%)	27 (82)	6(18)	0 (0)	0 (0)	0 (0)
Contact through web-based n, (%)	4 (12)	15 (46)	12 (36)	0	2 (6)

**Table 3:** Results for primary and secondary outcomes at baseline and 6-months follow-up for individuals with measurements at both time points

			Mean (SD)				
Variable	Group	n	Baseline	6 months	P-value^a^	Change^b^	P-value^c^
HbA1c (mmol/mol)	Intervention	33	63.5 (15.0)	62.8 (11.2)	0.66	-0.76 (9.9)	0.85
	Control	38	59.6 (11.5)	59.3 (11.7)	0.80	-0.34 (8.3)	
Time in range (%)	Intervention	30	45.3 (13.5)	50.8 (16.0)	0.08	5.50 (16.6)	0.34
	Control	34	51.1 (17.5)	52.6 (16.3)	0.62	1.47 (17.0)	
Time below range (%)	Intervention	30	8.0 (8.3)	7.0 (6.9)	0.57	-1.00 (9.6)	0.87
	Control	34	5.7 (4.6)	5.1 (4.3)	0.40	-0.68 (4.6)	
DTSQ, Hyper	Intervention	30	2.8 (1.4)	3.0 (1.3)	0.47	0.20 (1.3)	0.32
	Control	37	3.4 (1.3)	3.1 (1.6)	0.45	-0.27 (1.6)	
DTSQ, Hypo	Intervention	30	1.6 (1.1)	1.7 (1.3)	0.67	0.10 (1.3)	0.69
	Control	37	1.8 (1.3)	2.1 (1.3)	0.25	0.24 (1.6)	
DTSQ score	Intervention	29	29.9 (4.1)	30.5 (4.8)	0.36	0.62 (4.0)	0.75
	Control	37	28.8 (3.9)	28.8 (3.8)	0.68	-0.05 (4.2)	
Physical health	Intervention	30	67.6 (17.3)	68.3 (18.9)	0.48	0.70 (11.7)	0.57
	Control	37	68.1 (18.4)	69.7 (14.1)	0.73	1.59 (15.9)	
Physical burden	Intervention	30	10.5 (8.6)	7.3 (9.2)	**0.03**	-3.23 (9.6)	**0.02**
	Control	37	8.4 (9.2)	11.1 (8.1)	0.17	2.62 (10.0)	
Emotional health	Intervention	29	66.9 (21.1)	69.2 (19.5)	0.46	2.34 (18.2)	0.80
	Control	37	65.6 (16.3)	70.1 (15.9)	0.07	4.51 (16.1)	
Emotional burden	Intervention	29	9.5 (9.0)	6.9 (7.2)	0.08	-2.59 (10.9)	0.57
	Control	37	12.8 (10.5)	10.7 (10.9)	0.31	-2.11 (11.2)	
Social health	Intervention	29	79.9 (14.4)	78.1 (14.4)	0.37	-1.79 (16.0)	0.66
	Control	37	86.3 (16.9)	84.4 (14.5)	0.28	-1.86 (12.5)	
Social burden	Intervention	29	3.6 (7.6)	3.8 (8.2)	0.95	0.17 (5.3)	0.08
	Control	37	4.5 (9.9)	1.6 (4.0)	**0.01**	-2.92 (8.3)	
Quality of life	Intervention	28	73.0 (15.6)	76.1 (14.4)	0.30	3.11 (15.3)	0.70
	Control	36	77.1 (12.8)	78.2 (13.4)	0.44	1.08 (11.0)	
Burden quality of life	Intervention	27	11.3 (8.5)	11.0 (8.9)	0.44	-0.70 (6.3)	0.51
	Control	36	10.0 (8.7)	8.4 (8.5)	0.23	-1.61 (8.2)	

Note: Significant P-values are given in **bold**. DTSQ, Diabetes Treatment Satisfaction Questionnaire; SD, standard deviation, ^a^ P-value for test of differences between values at baseline and 6-months follow-up within groups. ^b^ Change from baseline to 6-months follow-up. ^c^ P-value for test of differences in change from baseline to 6-months follow-up between intervention and control groups

### Glycaemic control

The baseline HbA1c levels were comparable between the intervention group and the control group, with mean (± SD) values of 63.5 (± 15.0) mmol/mol and 59.6 (± 11.5) mmol/mol, respectively, and no significant differences. The same pattern was observed for the TIR (45.3% vs. 49.7%) and TBR (8.0% vs. 5.7%), again with no significant differences. At the 6-month follow-up, neither between-group nor within-group analyses revealed any significant differences in glycaemic control (HbA1c, TIR, TBR) at the 6-month follow-up. However, there was a numerical improvement by 5% units for TIR in the intervention group (p 0.08, Table 3).

### Perceived hyper- and hypoglycaemia

At baseline, there were no significant differences between the intervention and control groups in terms of perceived hyperglycaemia (2.8 ± 1.4 vs. 3.4 ± 1.3) or hypoglycaemia (1.6 ± 1.1 vs. 1.8 ± 1.3). After six months, no significant differences were observed within or between the groups for these parameters (Table 3).

### Diabetes treatment satisfaction

The study confirmed that there was no significant difference in diabetes treatment satisfaction between the intervention group (29.9 ± 4.1) and the control group (28.8 ± 3.9) at baseline, as measured by the DTSQ. Table 3 shows that there were no significant differences within or between the groups in terms of diabetes treatment satisfaction at the 6-month follow-up.

### Quality of life and check your health questionnaire

The study revealed that the intervention group demonstrated a significant decrease in the physical burden from baseline to 6 months. Thus, in the within-group analysis, the score decreased with 3.23 points, from a mean (± SD) score of 10.5 (± 8.6) points to 7.3 (9.2) points (p = 0.03). Moreover, the change in physical burden from baseline to 6 months differed significantly between the intervention and control groups (p = 0.02), with the score for the control group increasing with 2.62 points, from 8.4 (± 9.2) to 11.1 (± 8.1) points.

## Discussion

The present study found that the intervention had not significantly improved glycaemic control, perceived hyper- and hypoglycaemia, or diabetes treatment satisfaction compared with the control condition at the 6-month follow-up. In terms of this result, virtual diabetes care is thus neither better nor worse than usual care. Notably, the intervention did have a significant positive effect on the physical burden on quality of life.

### Results in perspective

The study results are consistent with those of prior research in the area of virtual health interventions for individuals with type 1 diabetes [15, 18]. One plausible explanation for the difficulties to reach optimal glycaemic control is that managing type 1 diabetes presents complex challenges both psychosocially and physically [24, 25] and the fear of developing late complications [26] requires complex management involving both physical and virtual meetings. Thus, virtual healthcare appointments may offer a complementary approach to traditional face-to-face care.

The present study included 79 participants, 35 in the intervention group and 44 in the control group. There were no significant differences between the two groups in terms of age, diabetes duration, sex, type of therapy or baseline HbA1c levels. At the 6-month follow-up, 71 participants were included. Regarding HbA1c levels and TIR, the participants in present study did not differ significantly from the group of young adults with diabetes type 1 with data in the Swedish National Diabetes Register. However, HbA1c levels were slightly higher among the participants in the present study, compared with data from the Swedish National Diabetes Register (63.5 vs. 59.6 mmol/mol).

The findings from this study suggest that while the intervention may not have had a significant impact on traditional diabetes outcomes such as glycaemic control, it did yield a favorable effect on a specific aspect of patient-reported outcome measures (PROM), specifically, the perceived burden of physical health. A potential explanation could be that the person can easily come into contact with the caregiver, which can create a feeling of security, which can lead to the person’s focus and energy being redirected towards other pursuits.

This is an important consideration for healthcare providers and policymakers when deciding on interventions for people with diabetes [3, 27, 28].

It is also noteworthy that both the intervention group and the control group perceived a high burden of living with diabetes, indicating that diabetes management is a complex and challenging task. Future research could explore additional interventions or support systems to alleviate the burden of diabetes management and improve quality of life for young people with diabetes type 1.

Overall, this study adds to the evidence base on diabetes management interventions and highlights the importance of considering PROMs such as quality of life alongside traditional clinical outcomes. Future research could further explore interventions that target non-traditional outcomes and investigate their long-term effects.

### Strengths and limitations

In addition to the findings discussed above, this study highlights the challenges of implementing interventions in real-world settings. Originally, our intention was to include 100 participants in the study. However, the widespread development of Covid-19 within society presented obstacles that hindered the recruitment of study subjects. The drop-out rate of 9.6% is not high for a clinical trial but does limit the generalisability of the findings. Furthermore, the study had a relatively short follow-up period of 6 months, and it is possible that longer-term follow-ups would reveal other outcomes.

The number of participants was limited, and some outcome differences may have been undetected. There was a skewed distribution in educational level between the randomized groups, and it was significantly lower among those participating in the intervention. Therefore, some positive effects of the intervention could have been underestimated.

Like any other questionnaire, the DTSQ has its limitations. One of these limitations is referred to as the ‘ceiling effect’. If a patient’s baseline DTSQ score is high enough, it may be challenging to detect any further improvements in treatment satisfaction following an intervention. Conversely, if the scores for Question 2 (‘hyperglycaemia’) or Question 3 (‘hypoglycaemia’) are low enough at baseline, it may be challenging to detect any decrease after intervention. This is an example of the ‘floor effect’.

Due to the vast array of technologies and applications included in digital-based interventions, it is challenging to draw broad conclusions from any single study. Despite numerous investigations into the effectiveness and usability of different tools and approaches, systematic reviews and meta-analyses have been unable to offer conclusive guidance because of variations in study designs, observation periods, study populations and the specific tools/technologies examined.

Despite these limitations, this study provides valuable information on the impact of the intervention on quality of life and suggests that interventions targeting non-traditional outcomes may have value. It is also worth noting that the intervention did not have any adverse effects, indicating that it is a safe option for people with diabetes.

## Conclusions

The implementation of a virtual diabetes clinic using the Vista Dialog platform did not result in significant improvements in glycaemic control or treatment satisfaction compared with usual care. However, it did show potential benefits in terms of reducing the burden on physical health and improving quality of life in young adults with type 1 diabetes. Further research is needed to explore the long-term effects and optimal use of virtual clinics in diabetes management.

## Data Availability

The datasets generated and/or analysed during the current study are not publicly available due to personal data protection legislation but are available from the corresponding author on reasonable request.

## List of abbreviations

BMI: Body mass index
CGM: Continuous glucose monitoring
isCGM: Continuous glucose monitoring with intermittent scanning/ flash glucose monitoring
CSII: Continuous subcutaneous insulin infusion
DTSQ: Diabetes Treatment Satisfaction Questionnaire
FGM: Flash Glucose Monitoring
HbA1c: Glycosylated haemoglobin
HCPs: Healthcare professionals
MDI: Multiple daily injections
PROM: Patient-reported outcome measure
RCT: Randomised controlled trial
rtCGM: real-time Continuous glucose monitoring/ Continuous glucose monitoring
SD: Standard deviation
TIR: Time in range
TBR: Time below range
